# 345. Dermatological Manifestations in Patients with COVID-19 Pneumonia in Veracruz, Mexico

**DOI:** 10.1093/ofid/ofab466.546

**Published:** 2021-12-04

**Authors:** Luis Del Carpio-Orantes, Sergio García-Méndez, Jesús Salvador Sánchez-Díaz, Andrés aguilar-silva, Elisa Estefania Aparicio-Sánchez, Sonia Cruz-Albarrán, Jorge Luis Denis-Bravo, Iván Esparza-Mora, Karem Samantha González-Medel, Nelyda Verania Hernández-Zaleta, Edgar Alberto López-Cruz, Laura Guadalupe Montano-Montiel, Jennifer Ivonne Morgado-Hernández, José Alberto Muñoz-Aguilar, Edson Irving Priego-Parra, Reynaldo Reich-Sierra, Noel Jhosimar Sánchez-Jiménez, María Fernanda Tress-Uc, Paola Velázquez-Orozco

**Affiliations:** 1 Instituto Mexicano del Seguro Social; Sociedad Mexicana de Virología, Veracruz, Veracruz-Llave, Mexico; 2 Hospital Regional de Alta Especialidad de Oaxaca, Oaxaca, Oaxaca, Mexico; 3 Instituto Mexicano del Seguro Social, Veracruz, Veracruz-Llave, Mexico

## Abstract

**Background:**

A large number of viral infections are characterized by the presence of cutaneous manifestations. Multiple dermatological manifestations have been observed in patients with COVID-19. Dermatological lesions in patients infected by SARS-CoV-2 such as livedo reticularis, rash and vascular lesions may represent manifestations of secondary phenomena such as paraviral rashes or by participation of the innate or adaptive immune system that cause vasodilation, vascular leakage or procoagulant effects

**Methods:**

Descriptive and observational study, adult patients with COVID-19 pneumonia were selected, confirmed by RT-PCR and chest CT. General symptoms, hematic cytometry results, pneumonia severity, prognosis as well as dermatological manifestations are characterized.

**Results:**

100 patients were entered into the study, with an average age of 49.4 years, 54% male. The general symptoms with the highest incidence were: fever, cough and dyspnea characteristic of SARS-CoV-2 infection, followed by chest pain, headache, anosmia and dysgeusia. The main alteration of the hemogram was lymphopenia, no leukopenia or plaquetopenia was demonstrated. 54% of those affected had mild pneumonia, the rest severe pneumonia. 75% progressed towards improvement and 25% died. Among the dermatological manifestations identified, all occurred in cases with severe pneumonia, the one with the highest incidence was the morbilliform viral exanthema in 18%, the presence of diffuse partial alopecia in 7% as well as manifestations of lividity and maceration in 1%. Regarding alopecia, in 6% it was reversible androgenetic alopecia, having manifested during the acute stage of pneumonia (all men), in 1% it presented alopecia areata (male) that has been persistent beyond the acute phase and in frank recovery

Demographic and clinical variables

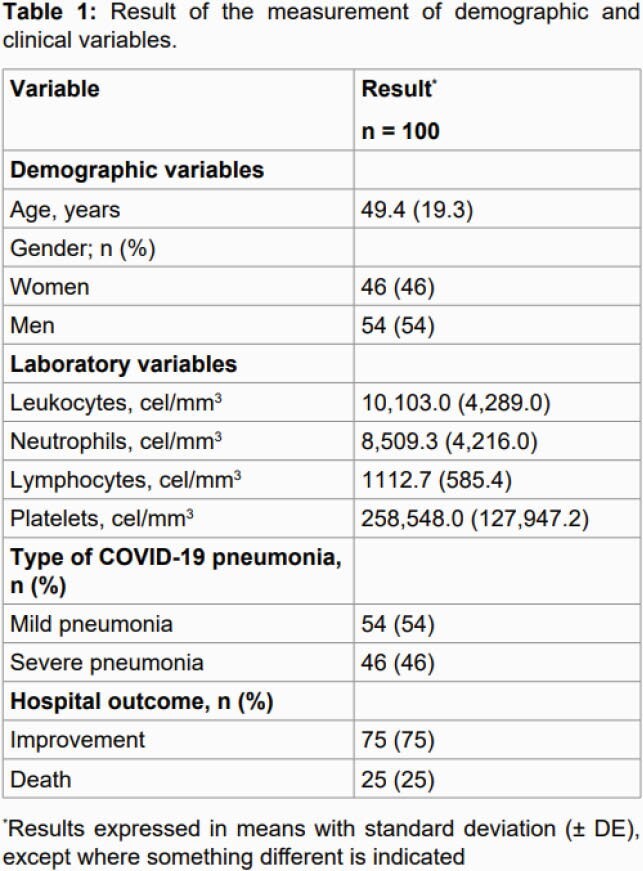

Clinical manifestations

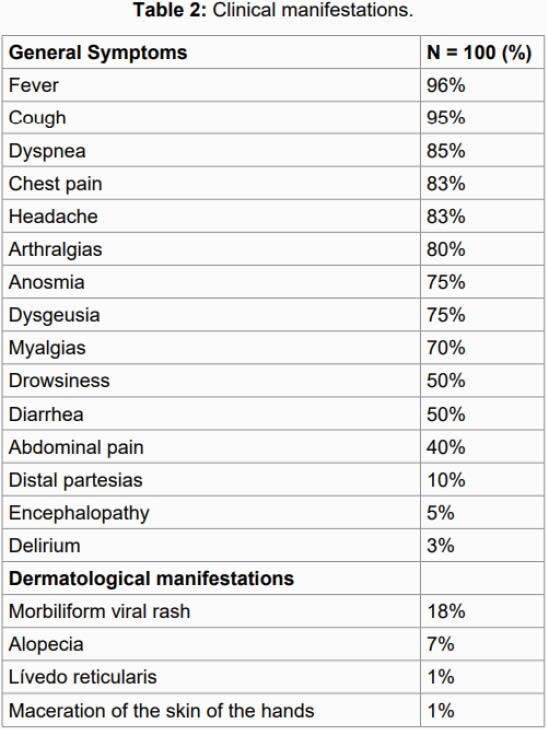

**Conclusion:**

The incidence of dermatological manifestations is low in this study population, the most frequent being the morbilliform viral exanthema expected in a virus, however they present manifestations of low incidence such as reversible androgenetic alopecia associated with severity of the disease, a finding that has been documented recently as a manifestation associated with COVID-19

**Disclosures:**

**All Authors**: No reported disclosures

